# Moving to the forefront of open access publishing in dermatology

**DOI:** 10.1002/ski2.3

**Published:** 2020-08-06

**Authors:** E.A. Langan

**Affiliations:** ^1^ Department of Dermatology University of Lübeck Lübeck Germany; ^2^ Dermatological Sciences University of Manchester Manchester UK

Whilst managing the coronavirus pandemic is rightly focussing the minds and efforts of the medical and research communities worldwide, open access (OA) publication and the launch of a new journal from the British Association of Dermatology (BAD), albeit the first in decades may seem barely worth a footnote.

However, precisely in this challenging and daily evolving context, the benefits of high‐quality, innovative, transparent, rapidly peer‐reviewed, and freely and widely accessible research is most tangible. The execution and publication of medical research in general, and in the case of *Skin Health and Disease* (*SHD*), dermatological research in particular, have to reflect and meet the demands of the 21st century scientific community. Indeed, OA publication is not only an attempt to improve the dissemination of key research discoveries, but it is potentially an invaluable tool to foster national and international research collaborations; of paramount importance to face global healthcare challenges.

To this end, *SHD* has a deliberately international focus, reflected in the number of nations represented on the Editorial Board. Moreover, George Millington, Editor‐in‐Chief, comes not only with a proven track record as a former editor of Clinical and Experimental Dermatology but also with a reputation for innovation.

My aim, as deputy editor, is to help support and shape the development of *SHD* and to ensure that the journal makes the most of the digital platform to facilitate engagement with the readership. Indeed, we will seek to actively engage both the scientific and lay community to become stakeholders in the development of the journal to ensure that *SHD* facilitates inter‐disciplinary research and involves the complete spectrum of researchers, from those at the start of their careers to renowned key world opinion leaders.

Starting this new venture, particularly in the context of a global viral pandemic, is going to be a challenge. However, with your support, and with the backing of our publisher, Wiley, *SHD* can be a standard bearer for the opportunities and advantages offered by OA publication and promises to establish itself as an indispensable member of the BAD publishing family.

## CONFLICT OF INTEREST

Ewan A. Langan has served on Advisory Boards for Novartis and Sun Pharma. In addition, Ewan A. Langan has received speakers' honoraria and/or meeting and travel support from Novartis, Curevac and BMS.

